# Neurosensory Deficits of the Mandibular Nerve Following Extraction of Impacted Lower Third Molars—A Retrospective Study

**DOI:** 10.3390/jcm12247661

**Published:** 2023-12-13

**Authors:** Marcus Rieder, Bernhard Remschmidt, Vera Schrempf, Matthäus Schwaiger, Norbert Jakse, Barbara Kirnbauer

**Affiliations:** 1 Division of Oral and Maxillofacial Surgery, Department of Dental Medicine and Oral Health, Medical University of Graz, 8036 Graz, Austria; marcus.rieder@medunigraz.at; 2 Division of Oral Surgery and Orthodontics, Department of Dental Medicine and Oral Health, Medical University of Graz, 8010 Graz, Austriamatthaeus.schwaiger@medunigraz.at (M.S.); norbert.jakse@medunigraz.at (N.J.); barbara.kirnbauer@medunigraz.at (B.K.)

**Keywords:** third molar extraction, neurosensory disturbance, wisdom tooth removal, oral surgery, mandibular nerve, inferior alveolar nerve, lingual nerve, neurosensory deficit

## Abstract

Background: Neurosensory deficits are one of the major complications after impacted lower third molar extraction leading to an impaired patient’s quality of life. This study aimed to evaluate the incidence of neurosensory deficits after lower third molar extraction and compare it radiologically to the corresponding position of the inferior alveolar nerve. Methods: In a retrospective study, all patients who underwent impacted lower third molar extraction between January and December 2019 were compiled. Therefore, clinical data as well as preoperative radiological imaging were assessed. Results: In total, 418 patients who underwent lower third molar extractions (*n* = 555) were included in this study. Of these, 33 (5.9%) had short-term (i.e., within the initial 7 postoperative days) and 12 (1.3%) long-term (i.e., persisting after 12 months) neurosensory deficits documented. The inferior alveolar nerve position in relation to the tooth roots showed apical position in 27%, buccal position in 30.8%, lingual position in 35.4%, and interradicular position in 6.9%. Conclusions: A statistically significant increased incidence of neurosensory deficits occurs when the inferior alveolar nerve is directly positioned lingually to the tooth roots (*p* = 0.01).

## 1. Introduction

The prevalence of individuals harboring at least one impacted tooth is documented to range between 18.8% and 40.5%, with lower third molars (LTMs) demonstrating the highest propensity for impaction [1,2,3,4,5]. Especially in people with a reduced gonial angle, and, consequently, a reduced retromolar space, there is a higher frequency of deeply impacted, horizontally positioned LTMs [6,7,8]. The extraction of LTMs represents one of the most frequently conducted procedures in the field of oral and maxillofacial surgery [9,10,11,12,13]. The indications for LTM removal encompass both therapeutic reasons, such as acute or chronic pericoronitis, cyst formation, non-restorable caries lesions, and prophylactic considerations [2,14]. Although there are rare complications, surgical site infections, pain, trismus, and three-dimensional measurable facial swelling are observed after LTM extraction [15]. However, by far the severest complication is postoperative neurosensory deficits. The anatomical variability and the position of LTMs in the posterior region of the alveolar crest near the inferior alveolar nerve (IAN) and the lingual nerve (LN) may result in a higher probability of complication occurrence compared to conventional tooth extractions, where these nerves are not in close proximity [16]. Damage to these structures is a substantial and quality-of-life-impacting complication that can arise after LTM removal [11,17]. This damage may occur due to a variety of factors, including injury to the neurovascular bundle during local anesthesia administration, as well as pressure exerted during tooth elevation or direct mechanical trauma to the nerve itself [18,19]. IAN deficits after LTM removal may manifest as hypesthesia, paresthesia, dysesthesia, or anesthesia affecting the lower lip, chin, buccal gingivae, and teeth on the affected side [20]. The documented incidence of complications to the IAN after LTM extraction exhibits a spectrum, ranging from 0.26% to 8.4% [11,21,22,23,24,25,26,27,28,29,30,31,32,33,34]. An injury to the LN, characterized by sensory deficits affecting the anterior two-thirds of the ipsilateral tongue and concurrent taste impairments, is reported with an incidence spanning from 0.1% to 22% [11,16,21,24,25,26,27,28,29,30,31,32,33,35]. However, it is imperative to distinguish between short-term and enduring sensory disturbances. Beyond the clinical evaluation of LTM, the incorporation of preoperative radiographic image analysis along with a careful surgical strategy is crucial and beneficial to prevent or minimize the aforementioned complications [36,37,38,39,40].

The aim of the current investigation was to ascertain the incidence of neurosensory deficits affecting the mandibular nerve (MN) after impacted LTM surgery, conducted at the Division of Oral Surgery and Orthodontics, Department of Dental Medicine and Oral Health, Medical University of Graz. In this study, particular attention was paid to assessing the relationship between the IAN and adjacent LTMs and distinguishing between short-term (7 days postoperatively) and long-term (at least 12 months postoperatively) sensory deficits.

## 2. Materials and Methods

First, a retrospective analysis was conducted based on preoperative radiological datasets and medical records of patients who underwent extraction of at least one impacted LTM in 2019 at the Department of Dental Medicine and Oral Health, Division of Oral Surgery and Orthodontics, Medical University of Graz. Exclusion criteria included preexisting neurosensory deficits of the MN prior to surgery and inadequate documentation. Second, a prospective evaluation of patients with postoperative neurosensory deficits was continued afterward.

The study was approved by the local ethics committee (IRB00002556, re: 33-093 ex 2012 and 33-575 ex 20/21). Patient consent was given from all participants of the clinical investigation.

All surgical procedures were executed following a standardized protocol at the University Clinic of Dental Medicine and Oral Health, Medical University Graz. First, local anesthesia was performed by a nerve block directed at the inferior alveolar nerve and the lingual nerve. In addition, depots were administered on the ascending mandible to anesthetize the buccal nerve, and submucosal depots were performed in the buccal region corresponding to teeth 37 and 47, respectively. The surgical access was made through an incision at the marginal gingiva of teeth 46 to 47, during which the dental papilla was detached. The incision was then extended from the distobuccal side of tooth 47 on the ascending mandible into the vestibule. Afterward, a freer was used to raise a full-thickness envelope flap. The retractor was employed to hold off the buccal portion of the flap, while a curved raspatorium was carefully inserted subperiosteal on the lingual side to ensure the preservation of the lingual nerve. The osteotomy was carried out with a rose bur until the crown of the tooth was completely exposed, and, if needed, the tooth was divided into pieces using a Lindemann bur. The removal of the tooth or the individual pieces was performed by means of a lever according to the leg or with surgical clamps. The wound was closed using non-absorbable sutures.

Data were analyzed from patient records and dental radiographs using the in-house computer systems Medocs (SAP, Walldorf, Germany) and Sidexis (version 4, Sirona Dentsply, Charlotte, NC, USA). All patients were examined one week after surgery, on the day of suture removal. Objective assessments including tests such as the light touch test, two-point discrimination threshold, pin-prick test, and vitality test of ipsilateral mandibular teeth were performed if subjective neurosensory deficits were reported by patients. The tests were conducted to evaluate the quality of neurosensory deficits (e.g., hypesthesia: light touch test, negative; two-point discrimination: negative; pin-prick test: positive; vitality test: positive). In cases of neurosensory deficits, follow-up examinations were conducted for a duration of 12 months after the suture removal. Incomplete recovery or persistent neurosensory deficits beyond 12 months of review were considered permanent. In cases where permanent deficits were observed, the Visual Analog Scale (VAS) ranging from zero to ten was employed to assess the pain impacting the quality of life.

Demographic data included age and sex. Radiological analysis was performed on the 2D panoramic radiographs (PR) datasets, as well as the standard dose 3D cone beam computed tomography (CBCT) datasets. PR scans were conducted using the Orthophos XG device (Dentsply Sirona, Bensheim, Germany). CBCT scans were conducted using either the Orthophos CBCT scanner (Dentsply Sirona, Bensheim, Germany) with the following parameters: 96 kV, 4.0 mA, an exposure time of 4.081 s, a field of view (FOV) of 10 × 5.9 cm, and a voxel size of 0.200 mm. Alternatively, the Planmeca ProMax 3D Max (Planmeca, Helsinki, Finland) with a field of view of 10.0 × 5.9 cm or 10.0 × 9.3 cm, covering a minimum of one complete dental arch, with a 200-mm voxel size (96 kV, 5.6–9.0 mA, 12 s) was employed.

The PR was used to evaluate the type of impaction (i.e., mesioangular, horizontal, distoangular, or vertical). CBCT scans were utilized to assess the positional alignment of the IAN in relation to the LTMs and to analyze their contact interactions. The position and the contact relation of the IAN relative to the roots of the LTM were defined according to Gu et al. and are provided below [36].

Class I: the mandibular canal is located on the apical side (apical position).
Class II: the mandibular canal is located on the buccal side (buccal position).
Class III: the mandibular canal is located on the lingual side (lingual position).
Class IV: the mandibular canal is located between the roots (interradicular position).

1. The mandibular third molar has no contact with the mandibular canal.
2. The mandibular third molar contacts with the mandibular canal with a complete white line.
3. The mandibular third molar contacts with the mandibular canal with a defective white line.
4. The mandibular third molar penetrates the mandibular canal.

Statistical analyses were performed using SPSS software (IBM SPSS statistics, version 27.0, IBM Corporation, Armonk, New York, NY, USA) with a 5% significance level. Chi-square tests were used for quantitative analyses. Fisher’s exact tests and Chi-square tests were used to analyze categorical data. Independent Student’s *t*-tests were applied to continuous variables.

## 3. Results

### 3.1. Incidence of Neurosensory Deficits

A total of 418 patients (*n* = 418) who underwent the surgical removal of their LTMs (*n* = 555) were included. Of these patients, 58% were female (*n* = 241), and 42% were male (*n* = 177). The age of the participants ranged from 15 to 93 years, with a mean age of 29.1 ± 11.2 years. In 51.2% of cases, the left LTM was extracted, while in 48.8% of cases, the right LTM was removed. A majority of the surgeries (*n* = 399, 70.1%) were carried out by postgraduate dentists or maxillofacial surgeons, while dentistry students conducted the remaining 29.9% of the operations (*n* = 166).

The overall incidence of acute neurosensory deficits of the MN (inferior alveolar nerve and/or lingual nerve) within the first 7 days after extraction of the LTM amounted to 5.9% (33/555). Among these cases, the inferior alveolar nerve (IAN) was affected in 2.9% (*n* = 16), while the lingual nerve (LN) was impaired in 2.2% (*n* = 12), with 0.5% (*n* = 3) experiencing combined deficits. Additionally, 0.4% of cases had an unknown area of affection. The predominant neurosensory deficit was an IAN impairment, constituting 48.5% of cases (*n* = 16), closely followed by isolated LN deficits at 36.4% (*n* = 12). The occurrence of a simultaneous IAN and LN disturbance was rare and observed in only 9.1% of cases (*n* = 3). In two instances (*n* = 2), a neurosensory deficit was recorded in our electronic database; however, detailed information regarding the specific location of the affected nerve was lacking.

**Figure 1:**
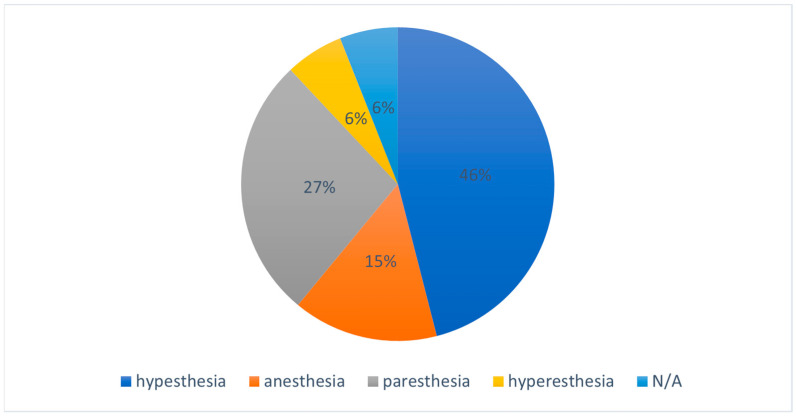
Quality of nerve disorder.

The documented characteristics of nerve deficits encompassed hypesthesia (45.4%; *n* = 15), paresthesia (27.3%; *n* = 9), anesthesia (15.1%; *n* = 5), and hyperesthesia (6.1%; *n* = 2) (Figure 1). As previously noted, detailed information on the nature of neurosensory deficits was absent in two cases. Notably, dysesthesia was not observed in any of the cases. The presence of hyperesthetic disturbances displayed statistical significance (*p =* 0.006, df = 3, χ^2^ = 12.3).

### 3.2. Type of Impaction

Using the standardized PRs, it was observed that 14.4% (*n* = 80) of the LTMs exhibited no angulation (vertical position). Mesioangular orientation was prevalent in 39.1% (*n* = 217) of cases, while 18.2% (*n* = 101) displayed a distoangular orientation. Furthermore, horizontal angulation was documented in 26.7% (*n* = 148) of the studied cases (Table 1). No statistical significance was observed concerning the association between the pattern of impaction and neurosensory deficits (*p* = 0.613, df = 3, χ^2^ = 1.72).

### 3.3. Position of the Mandibular Canal Relative to the Apex

The routine practice in our clinic does not involve the standard performance of a CBCT scan prior to LTM removal. Rather, we employ such scans selectively, guided by radiographic indicators within the PR suggesting an elevated risk of nerve injury during the extraction of the corresponding tooth. These indicators include instances of suspected contact or overlapping of structures between the LTM and the mandibular canal, alongside instances involving complex tooth anatomy or cystic lesions.

A preoperative CBCT scan was conducted in 47.2% of the surgical procedures (*n* = 263). Within this subset of images, the position of the IAN in relation to the tooth roots was assessed. The IAN showed an apical position relative to the tooth roots in 27% of cases (*n* = 71), a buccal position in 30.8% of cases (*n* = 81), a lingual position in 35.4% of cases (*n* = 93), and an interradicular position in 6.9% of cases (*n* = 18).

**Figure 2:**
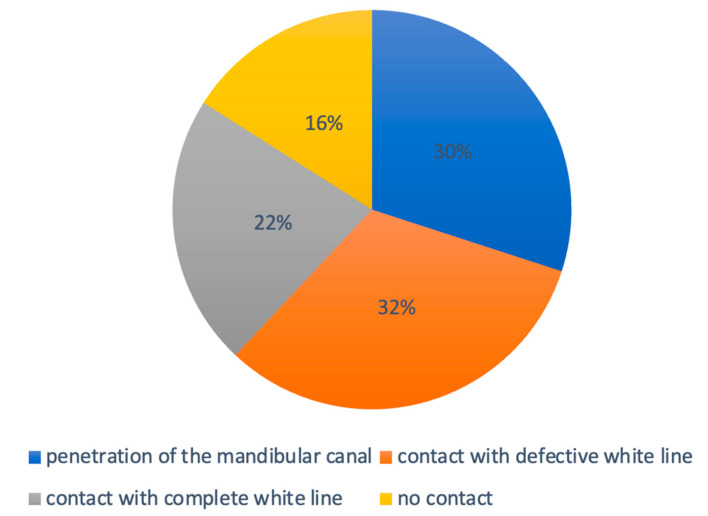
Contact relation of the inferior alveolar nerve canal with the wisdom tooth roots.

**Figure 3:**
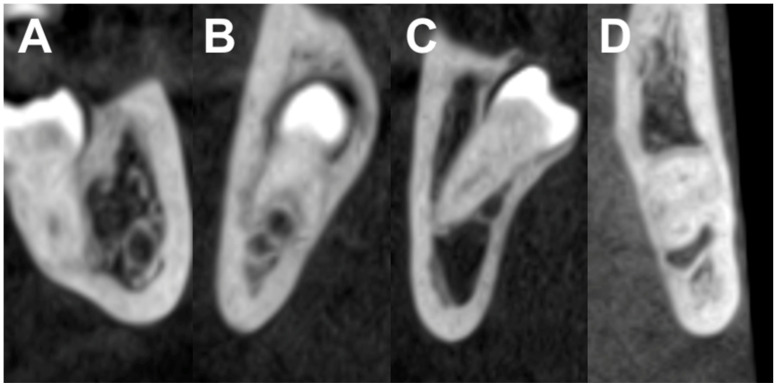
CBCT images illustrate the anatomical relationship between the mandibular canal and the roots of a lower third molar. (**A**) Class II/buccal: No contact; (**B**) Class IV/interradicular: Contact with a complete white line; (**C**) Class III/lingual: Contact with a defective white line; (**D**) Class I/apical: Penetration of the mandibular canal.

There was a notable predominance of LTM having direct contact with the IAN (84%, *n* = 221), whereas 16% (*n* = 42) of the third molars did not demonstrate this direct contact. In 21.7% (*n* = 57) the lower third molar contacted with the mandibular canal with a complete radiopaque boundary, in 32.3% (*n* = 85) with an interrupted radiopaque boundary, and in 30.4% (*n* = 80) the wisdom tooth penetrated the nerve canal (Figure 2 and Figure 3).

**Figure 4:**
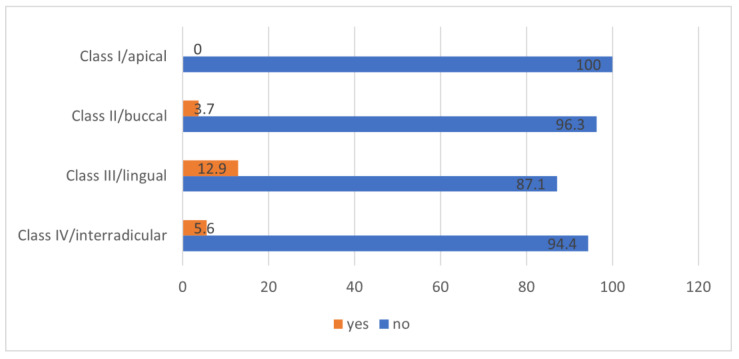
Relationship between the inferior alveolar nerve position and the lower third molar roots, and the occurrence of neurosensory deficits.

The incidence of IAN neurosensory deficits and the associated position of the roots of LTMs relative to the IAN were analyzed in LTMs with an available CBCT scan (16/263). It was found that those IANs having an apical position relative to the roots of LTMs, had an incidence of 0% of postoperative neurosensory disturbances. Buccal position resulted in 3.7% (3/81), interradicular position in 5.6% (1/18), and lingual position in 12.9% (12/93) in an acute postoperative neurosensory deficit. Nerve disturbance was significantly more frequent in the IANs having a lingual position relative to the roots of LTMs (*p =* 0.01, df = 4, χ^2^ = 13.1) (Figure 4).

**Figure 5:**
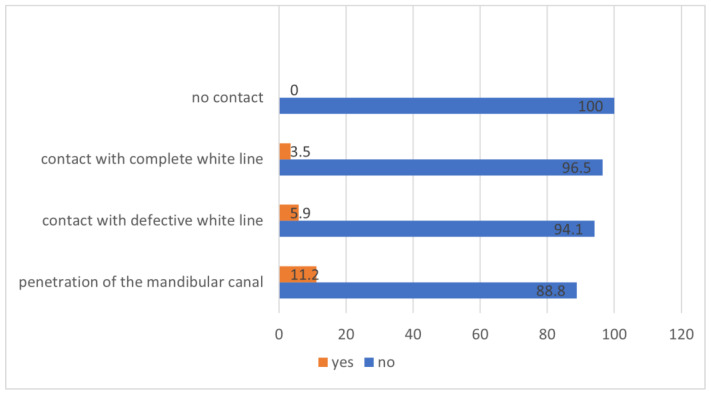
Contact relation and occurrence of neurosensory disorders (%).

There was no case of neurosensory deficit when there was no contact between the LTM and the IAN (0/42) and an occurrence of 7.2% (16/221) when there was contact. However, no statistical significance could be found (*p* = 0.061, df = 1, χ^2^ = 3.15). Analyzing the exact contact relationships, it was noted that among the cases of nerve injuries observed, 3.5% (*n* = 2) involved the IAN making direct contact with the apex with a complete radiopaque boundary. In 5.9% (*n* = 5) of instances there was a contact with a defective radiopaque boundary and in 11.3% (*n* = 9) of the cases, the LTM exhibited a penetration of the mandibular canal. However, there was no statistically significant difference between the contact relation and the occurrence of neurosensory deficits (*p* = 0.070, df = 3; χ^2^ = 7.06) (Figure 5).

### 3.4. Sex and Age

The incidence of acute postoperative neurosensory deficit of the MN in males and females was 4.6% (10/218) and 6.8% (23/337), respectively. No statistically significant difference in gender distribution was observed with respect to nerve disturbances (*p* = 0.183, df = 1, χ^2^ = 1.85). The mean ages of patients who experienced MN injury were 29 ± 6.9 years old, and this was not significantly different from the mean age of 29.1 ± 11.4 years in patients who showed uneventful healing (*p* = 0.967, mean diff = −0.08; 95% CI [−4.94; 3.87]).

### 3.5. Experience of Operators

Among the surgical procedures executed by postgraduate dentists or maxillofacial surgeons, 5.9% (23/389) resulted in postoperative neurosensory deficits. Conversely, in the surgeries conducted by dental students, the occurrence rate was 6% (10/166). There was no statistically significant difference in the incidence of neurosensory deficits between the patients operated on by students and postgraduate doctors/maxillofacial surgeons (*p* = 0.548, df = 1, χ^2^ = 0.003).

### 3.6. Recovery Patterns

A total of 14 out of 33 patients who experienced IAN or LN deficits within the initial seven postoperative days did not attend any follow-up appointments after the first postoperative review. This resulted in a drop-out rate of 42.4%. The follow-up period extended over 12 months, with the number of visits varying according to the performed therapy regime and therapy response.

In this study, the incidence of persisting neurosensory deficits of the mandibular nerve stood at 1.3% out of 555 removed teeth. Specifically, the IAN was affected in 0.8% of cases (*n* = 5) and the LN was affected in 0.5% of cases (*n* = 3), with no statistically significant difference detected (*p =* 0.705, df = 1, χ^2^ = 0.14). In the cohort of patients with enduring nerve deficits, 71.4% (*n* = 5) exhibited hypoesthesia in the affected area, while 28.6% (*n* = 2) experienced paresthesia. No other sensory qualities were identified, and this outcome did not demonstrate statistical significance (*p =* 0.257, df = 1, χ^2^ = 1.29). Employing a VAS, it was observed that none of the affected patients (0/7) exhibited permanent pain reducing their quality of life.

#### Therapy Regime

Five different therapeutic regimens were employed to treat acute postoperative neurosensory deficits, and the process of recovery was evaluated through objective assessments, including the light touch test, pin-prick test, and vitality test of the teeth on the affected side:

1. The combination of cortisone (prednisolone 5mg), vitamin B-complex, and low-level laser therapy was used in 26.3% (*n* = 5) of the cases and had a complete recovery in 60% (3/5).
2. The combination of vitamin B-complex and low-level laser therapy in 21.1% (*n* = 4) with a recovery rate of 50% (2/4).
3. Only low-level laser therapy in 15.8% (*n* = 3) with a recovery rate of 100% (3/3).
4. The combination of cortisone and vitamin B-complex in 5.3% (*n* = 1) with a recovery rate of 0% (0/1).
5. Only vitamin-b-complex in 21.1% (*n* = 4) with a recovery rate of 75% (3/4).

## 4. Discussion

According to the literature, the frequency of acute MN neurosensory deficit after removal of the LTM ranges from 1% to 16.3% [10,11,12,16,41,42,43,44,45,46]. In our study, the incidence of acute nerve deficits was 5.9% and is thereby consistent with results reported in the literature. According to Akashi et al., the majority of the affected patients suffer from hyperesthesia [10]. Concerning nerve deficits among the included patients, the IAN was affected in 2.9%, the LN in 2.2%, and a combination of both in 0.5%. The incidence of permanent neurosensory disorders of the MN after LTM surgery is reported to be approximately 1% in the majority of studies found in the literature [11,42,44,46,47,48,49]. Our study is also consistent with these findings, showing an incidence of 1.3%. Breaking down the permanent nerve deficits, the IAN was damaged in 0.8% of cases and the LN in 0.5% of cases.

Several authors found a correlation between age and a higher susceptibility to nerve disturbances following impacted LTM extractions. Bruce et al. demonstrated a significantly elevated risk of nerve deficits in patients aged 35 years or older compared to younger patients [25]. This result corresponds with the observations made by Blondeau et Daniel, who proposed that this association might be attributed to increased bone density, reduced bone elasticity, diminished healing capacity, and the completion of root formation [9]. Nevertheless, some studies refute a link between age and the risk of neurosensory deficits [11,24,28,50,51,52]. The findings of the current study also do not provide evidence to support the hypothesis that age elevates the risk of MN deficits (*p* = 0.967). Furthermore, as with most studies in the literature, we found no association between the gender of the participants and the incidence of nerve deficits.

The level of the surgeon’s experience performing the removal of the impacted LTM has often been considered a potential risk factor for nerve deficits. Sisk et al. postulated that the likelihood of developing neurosensory deficits increases with the surgeon’s lack of experience [34]. Similarly, Cheung et al. observed and reported 33 of 45 mandibular nerve deficits when a dental student performed the surgeries [11]. In contrast to the aforementioned results, our study did not find a significant association between surgeon experience and the risk of neurosensory deficits. However, this lack of association could possibly be due to the fact that more challenging cases in our department were assigned to dentists with more experience. Furthermore, students were at the operations under the strict supervision of experienced surgeons.

Analysis of the standardized PRs of all LTMs with reference to the angulation found a mesioangular impaction in 39.1% being the most frequent angulation, followed by a horizontal angulation in 26.7%. Our results report the highest incidence of nerve deficits in mesial angulated LTMs (42.4%). However, no statistical significance was found. Barry et al. showed a similar frequency distribution regarding angulation in their study but could not present a significant result of the association between angulation and nerve deficits [43]. Shiratori et al. found horizontal angulation to be the most common and described horizontally angulated LTM as having the highest risk of nerve deficits. However, no statistically significant result was found, which is in accordance with the current study [53].

In the case of 263 LTMs, we obtained a CBCT scan preoperatively. Analyzing the scans, we found the lingual position of the IAN relative to the roots to be the most frequent one (35.4%). This finding is contrary to the results of the study by Gu et al., where an apical position was observed in 88.1% of cases [36]. The difference and nearly uniform distribution of the IAN position to the root in our study may be due to the fact that when the PR showed apical root positions without contact with the IAN, no routine CBCT scans were performed.

Despite the lack of statistical significance, our study revealed a pattern in which acute postoperative neurosensory deficits manifested exclusively in patients whose LTMs were in direct contact with the IAN (*p* = 0.061). Lingually located nerves had a significantly higher risk of postoperative deficits, with a statistically significant result (*p* = 0.011). While Shiratori et al. reported comparable results, Barry et al., conversely, found an increased risk of neurosensory deficits when the nerve was located buccal or interradicular to the roots of LTMs [43,53]. According to several authors, the contact relation of the LTM and the IAN seems to be a more important nerve deficit risk predictor than the position itself [41,45,53]. It needs to be mentioned that our investigation did not identify a statistically significant difference in the incidence of IAN deficits, regardless of whether the contact between the roots and the IAN was demarcated by a complete radiopaque boundary, a defective radiopaque boundary, or a penetrating nerve (*p* = 0.07).

As mentioned, there were short-term neurosensory deficits at the LN in 2.2% and long-term deficits in 0.5% of the cases. In our department, a lingually inserted raspatorium is used to enhance visibility and protect the soft tissue. According to a meta-analysis, the incidence of transient nerve damage to the LN varies depending on the surgical technique, namely without lingual flap (1.24%), with lingual flap (2.39%), and with lingual split technique (2.44%). With regard to permanent damage, the study could not find any advantage for the use of lingual flaps or the lingual split technique [54].

Usually, neurosensory deficits recover spontaneously within the first 6 months after surgery [11,19,55,56,57,58]. However, a major challenge in the management of these neurosensory deficits is the lack of standardized treatment protocols [19]. Interestingly, it was observed that none of the patients with persistent neurosensory deficits (i.e., 12 deficits after 12 months) reported a decreased quality of life. It can be assumed that this phenomenon is due to a possible habituation effect.

Several limitations must be acknowledged in this study. First, the retrospective study design inherently carries the risk of selection bias and uncontrolled variables. Second, the absence of a standardized treatment regimen may introduce variability in patient outcomes. For example, the administration of drugs such as glucocorticoids before and after surgery could have affected the development of sensorimotor disorders. Third, the lack of standardized follow-up procedures hinders our ability to comprehensively assess the efficacy of individual treatments. Additionally, the high drop-out rate during follow-up, accounting for 42.4% of the initial cohort, poses challenges in drawing firm conclusions. In addition, there is a possibility that the MN was injured preoperatively during the administration of the nerve block. Unfortunately, the removal of LMTs near the nerve without a nerve block seems clinically impossible. To address these limitations, future studies should consider adopting a multicenter approach with well-defined and standardized treatment and follow-up regimens, thereby enhancing the reliability and generalizability of the findings.

## 5. Conclusions

The occurrence of neurosensory deficits at the MN after LTM surgery is relatively rare. Our results are consistent with the majority of published studies found. A lingual position of the IAN in close proximity to the LTM significantly increases the risk of nerve deficits. In this context, the use of CBCT scans appears promising as it can improve risk assessment and provide comprehensive preoperative patient information. It is noteworthy that in our study no decreased quality of life was observed in patients with persistent nerve deficits.

## Figures and Tables

**Table 1:** Pattern of impaction.

Angulation	*n*	Neurosensory Deficits
vertical	80 (14.4%)	5 (6.3%)
mesioangular	217 (39.1%)	14 (6.5%)
distoangular	101 (18.2%)	8 (7.9%)
horizontal	148 (26.7%)	6 (4.1%)

## Data Availability

The data presented in this study are available on request from the corresponding author. The data are not publicly available due to privacy restrictions.
